# Editorial: Posterior urethral valves: advances in diagnosis, management, and long-term follow up

**DOI:** 10.3389/fped.2023.1252048

**Published:** 2023-07-19

**Authors:** Massimo Garriboli, Joanna Clothier, Giorgio Selvaggio, Luke Harper

**Affiliations:** ^1^ Paediatric Urology, Evelina London Children’s Hospital, London, United Kingdom; ^2^ Stem Cells & Regenerative Medicine Section, Developmental Biology & Cancer Programme, UCL Institute of Child Health, London, United Kingdom; ^3^ Paediatric Nephrology and Bladder Service, Evelina London Children’s Hospital, London, United Kingdom; ^4^ Department of Paediatric Surgery, V. Buzzi Children’s Hospital, Milan, Italy; ^5^ Department of Paediatric Urology, Centre Hospitalier Universitaire de Bordeaux, Bordeaux, France

**Keywords:** posterior urethral valves (PUV), long-term follow up, diagnosis, paediatric urology, management - healthcare

Editorial on the Research Topic Posterior urethral valves: advances in diagnosis, management, and long-term follow up

Posterior Urethral Valves (PUV) is a complex and fascinating condition that affects the urinary tract in boys. It represents the most common cause of congenital urinary outflow obstruction affecting approximately 1 in 3,000 to 8,000 male births (1).

Despite many recent advances in the understanding of PUV, there is still much to discover on the causes, evolution, and management of this condition, from the embryo up to adulthood. Van der Zanden et al. reviewed the files of more than 1,000 children (407 PUV and 814 controls) exploring potential maternal risk factors and identified that a family history of congenital anomalies of the kidneys and urinary tract (CAKUT) and maternal pre-existing hypertension as well as young maternal age seem to be associated with a higher risk of PUV development. This finding is in contrast with other reports that link increased maternal age (>35 years) as a risk factor for chromosomal anomalies (2) or urogenital defects, as shown by the recent systematic review published by Ahn et al. (3).

The quest for identifying genetic profiles and associated congenital defects is open and PUV is definitely an interesting condition to investigate (4). The association of PUV with genetic defects such as trisomy 21 has already been shown (5), but a more advanced exercise to explore the genetic association with PUV is represented by genome-wide association studies (GWAS). In 2022, Chan et al. identified TBX5 (T-Box Transcription Factor 5) and PTK7 (Protein Tyrosine Kinase 7) located in chromosomes 12 and 6, respectively, as candidate genes associated with PUV (6). Another GWAS for PUV, as well as a meta-analysis, was performed by van der Zanden et al. in four cohorts of patients and controls. They identified three suggestive loci in chromosomes 13, 16, and 20 but eventually found no definitive association, suggesting that more studies are needed to enable the identification of any possible significant association.

Over the years, the survival rate of foetuses with PUV has increased significantly. However, a large proportion of boys born with PUV will still face a degree of bladder and renal deterioration and require multiple hospital admissions and interventions including, for the most severe cases, renal replacement. Multiple reports have previously suggested that nadir serum Creatinine is an accurate prognostic factor for mid and long-term renal function (7).

The meta-analysis performed by Meneghesso et al. reinforces this idea and they suggest a cut-off of 1 mg/dl to be significant.

The bladder represents a crucial player in the long-term outcome of boys born with PUV and this is being recognized more and more by clinicians and researchers, thus highlighting the need for more thorough investigations of its function, both in the short and long term. The paper from Pellegrino et al. shares their 30-year experience with PUV management and highlights the role of the bladder, including the association with Vesico-ureteric reflux (VUR) and Bladder neck dysfunction (BND), as well as the importance of long-term follow-up.

It must be said that the question of whether VUR and BND are both primary conditions associated with a structural anomaly of the urinary tract development or whether they are secondary to the bladder maldevelopment and caused by the high pressures developed during voiding and thickening of the detrusor muscle is both unanswered and fascinating (8).

On another level, the complex relationship between bladder pressure and renal function in the presence of venting mechanisms known as “pressure pop-off mechanisms” has been investigated for many years since the original description by Rittenberg et al. (9). More and more studies, however, have challenged the original belief that they would have a protective effect on the renal function and some teams have suggested that the presence of pop-off could actually represent a negative prognostic factor (10). The retrospective review published by Khondker et al. suggested no association between VURD and bladder dysfunction at 120 months of follow-up (11). In the study by Delefortrie et al., in a series of 137 PUV patients with a minimum of 5 years follow-up, the investigators found no significant difference in the mean renal function between boys with and without pop-off. They do, however, specifically question whether patients with and without pop-off mechanisms are truly comparable as we do not know why in the presence of high-pressure some will present a pop-off mechanism and why if they do, it will be in the form of VURD, urinoma, or a diverticula.

One of the factors that influence the long-term renal function in boys born with PUV is represented by the development of febrile urinary tract infections (fUTIs). The association between fUTIs and VUR is well known and a link with a higher risk of developing renal scarring has been clearly demonstrated (12).

In their secondary analysis of a multicentre randomised control trial, Harper et al. demonstrated this association in boys with PUV. Their data showed that the presence of high-grade VUR is associated with a 6-fold higher risk of presenting fUTIs, and, interestingly, they also identified the first 9 months of life as the more critical period in which boys have a higher risk of developing a febrile UTI. Circumcision plays a crucial role in mitigating the above risk and is, therefore, recommended, particularly in prenatally diagnosed cases (13).

It would be interesting to explore many other aspects of the long-term outcomes of boys born with PUV, including boys' psychological development, quality of life, and life achievements.

The complexity and low incidence of the condition make long-term outcomes of PUV scarcely reported in the literature, with most publications either reporting small series or including a very large time span (14).

The efforts of clinicians and researchers should therefore be focussed on identifying common guidelines and agreed pathways that will enable them to join and compare short and long-term results and enhance the quality of care for boys born with PUV.

## References

[1] Thakkar D Deshpande AV Kennedy SE. Epidemiology and demography of recently diagnosed cases of posterior urethral valves. Pediatr Res. (2015) 76:560–3. 10.1038/pr.2014.134 25198372

[2] Aprigio J de Castro CML Lima MAC Ribeiro MG Orioli IM Amorim MR. Mothers of children with down syndrome: a clinical and epidemiological study. J Community Genet. (2023) 14(2):189–95. 10.1007/s12687-022-00627-7 36562914 PMC10104982

[3] Ahn D Kim J Kang J Kim YH Kim K. Congenital anomalies and maternal age: a systematic review and meta-analysis of observational studies. Acta Obstet Gynecol Scand. (2022) 101(5):484–98. 10.1111/aogs.14339 35288928 PMC9564554

[4] Silvén H Savukoski SM Pesonen P Pukkala E Ojaniemi M Gissler M Association of genetic disorders and congenital malformations with premature ovarian insufficiency: a nationwide register-based study. Hum Reprod. (2023) 38(6):1224–30. 10.1093/humrep/dead066 37018629 PMC10233236

[5] Garriboli M Ibrahim S Clothier J. Spontaneous bladder rupture secondary to posterior urethral valves in a boy with down syndrome. BMJ Case Rep. (2021) 14(9):e240857. 10.1136/bcr-2020-240857 34551910 PMC8461274

[6] Chan MM Sadeghi-Alavijeh O Lopes FM Hilger AC Stanescu HC Voinescu CD Diverse ancestry whole-genome sequencing association study identifies TBX5 and PTK7 as susceptibility genes for posterior urethral valves. Elife. (2022) 11:e74777. 10.7554/eLife.74777 36124557 PMC9512401

[7] Wu CQ Blum ES Patil D Shin HS Smith EA. Predicting childhood chronic kidney disease severity in infants with posterior urethral valve: a critical analysis of creatinine values in the first year of life. Pediatr Nephrol. (2022) 37(6):1339–45. 10.1007/s00467-021-05271-w 34716802

[8] Holmdahl G. Bladder dysfunction in boys with posterior urethral valves. Scand J Urol Nephrol Suppl. (1997) 188:1–36. PMID: 9458522

[9] Rittenberg MH Hulbert WC Snyder HM 3rd Duckett JW. Protective factors in posterior urethral valves. J Urol. (1988) 140(5):993–6. 10.1016/S0022-5347(17)41908-2 3139895

[10] Paraboschi I Giannettoni A Mantica G Polymeropoulos A Mishra P Clothier J Posterior urethral valves, unilateral vesicoureteral reflux, and renal dysplasia (VURD) syndrome: long-term longitudinal evaluation of the kidney function. Int J Environ Res Public Health. (2023) 20:6238. 10.3390/ijerph20136238 37444086 PMC10341772

[11] Khondker A Yadav P Kim JK Chua ME Brownrigg N Richter J Does VURD syndrome impact voiding efficiency in posterior urethral valves? J Pediatr Urol. (2023):S1477-5131(23)00109-2. 10.1016/j.jpurol.2023.03.022 37019713

[12] Keren R Shaikh N Pohl H Gravens-Mueller L Ivanova A Zaoutis L Risk factors for recurrent urinary tract infection and renal scarring. Pediatrics. (2015) 136(1):e13–21. 10.1542/peds.2015-0409 26055855 PMC4485012

[13] Harper L Blanc T Peycelon M Leclair MD Garnier S Flaum V Circumcision and risk of febrile urinary tract infection in boys with posterior urethral valves: result of the CIRCUP randomized trial. Eur Urol. (2021) 22:64–72. 10.1016/j.eururo.2021.08.024 34563412

[14] Lopez Pereira P Martinez Urrutia MJ Espinosa L Jaureguizar E. Long-term consequences of posterior urethral valves. J Pediatr Urol. (2013) 9(5):590–6. 10.1016/j.jpurol.2013.06.007 23871421

